# Deficiency of *Cks1* Leads to Learning and Long-Term Memory Defects and p27 Dependent Formation of Neuronal Cofilin Aggregates

**DOI:** 10.1093/cercor/bhw354

**Published:** 2016-11-19

**Authors:** Alexander Kukalev, Yiu-Ming Ng, Limei Ju, Amal Saidi, Sophie Lane, Angeles Mondragon, Dirk Dormann, Sophie E. Walker, William Grey, Philip Wing-Lok Ho, David N. Stephens, Antony M. Carr, Karri Lamsa, Eric Tse, Veronica P. C. C. Yu

**Affiliations:** 1 Eukaryotic Chromatin Dynamics Group , MRC Clinical Sciences Centre , Imperial College Hammersmith Campus , London W12 0NN , UK; 2 Department of Medical and Molecular Genetics , King's College London School of Medicine , Guy's Hospital , Great Maze Pond , London SE1 9RT , UK; 3 Division of Haematology , Department of Medicine , The University of Hong Kong , Hong Kong; 4 Genome Damage and Stability Centre , School of Life Sciences , University of Sussex , Falmer, Sussex BN1 9RQ , UK; 5 Microscopy Facility , MRC Clinical Sciences Centre , Imperial College Hammersmith Campus , London W12 0NN , UK; 6 School of Psychology , University of Sussex , Sussex, Brighton BN1 9QG , UK; 7 Division of Neurology , Department of Medicine , University of Hong Kong , Hong Kong; 8 Department of Pharmacology , Oxford University , Oxford OX1 3QT , UK; 9 Current address: Epigenetic Regulation and Chromatin Architecture Group , Berlin Institute for Medical Systems Biology, Max-Delbrück Centre for Molecular Medicine , Robert-Rössle Strasse , Berlin-Buch 13125 , Germany; 10 Current address: Department of Physiology, Anatomy and Neuroscience , University of Szeged , Közép fasor 52 , Szeged H-6726,Hungary

**Keywords:** cyclin-dependent kinase, hippocampus, long-term potentiation, RhoA, synaptic plasticity

## Abstract

In mitotic cells, the cyclin-dependent kinase (CDK) subunit protein CKS1 regulates S phase entry by mediating degradation of the CDK inhibitor p27. Although mature neurons lack mitotic CDKs, we found that CKS1 was actively expressed in post-mitotic neurons of the adult hippocampus. Interestingly, *Cks1* knockout ( *Cks1*^*−/−*^ ) mice exhibited poor long-term memory, and diminished maintenance of long-term potentiation in the hippocampal circuits. Furthermore, there was neuronal accumulation of cofilin-actin rods or cofilin aggregates, which are associated with defective dendritic spine maturation and synaptic loss. We further demonstrated that it was the increased p27 level that activated cofilin by suppressing the RhoA kinase-mediated inhibitory phosphorylation of cofilin, resulting in the formation of cofilin aggregates in the *Cks1*^−/−^ neuronal cells. Consistent with reports that the peptidyl-prolyl-isomerase PIN1 competes with CKS1 for p27 binding, we found that inhibition of PIN1 diminished the formation of cofilin aggregates through decreasing p27 levels, thereby activating RhoA and increasing cofilin phosphorylation. Our results revealed that CKS1 is involved in normal glutamatergic synapse development and dendritic spine maturation in adult hippocampus through modulating p27 stability.

## Introduction

The establishment of long-term memory requires structural plasticity of dendritic spines, which contributes to altered synaptic strength between neurons. Recent studies have begun to unravel an increasing number of molecular partners that underscore this process ( Rochefort and Konnerth 2012 ), some of which are also involved in non-neuronal cellular processes. The functions of these molecules range from sculpting the cytoskeleton during mitosis and cellular migration, to control of the cell cycle ( Odajima et al. 2011 ).

CKS1 was first identified as a cyclin-dependent kinase (CDK) interacting protein that participates in cell-cycle control ( Pines 1996 ). In the mitotic cell cycle, mammalian CKS1 binds CDK1 and CDK2. Expression of *CKS1* is particularly elevated in many aggressive human cancers, where its oncogenic role has been ascribed to a second function for the protein as a cofactor to the ubiquitin ligase complex, SCFSKP2 ( Hao et al. 2005 ). SKP2 ubiquitylates and therefore mediates proteasomal-dependent degradation of a number of important substrates that control G1—S phase transition of the cell cycle. This includes the CDK inhibitor, p27. In mice knocked down for *Cks1* , high levels of p27 accumulate ( Spruck et al. 2001 ). Another level of post-translational regulation of p27 level is mediated by peptidyl-prolyl-isomerase PIN1. PIN1 binds specifically to its substrates at the serine/threonine-proline motifs when the serine or threonine residue is phosphorylated, and catalyzes isomerization of serine/threonine-proline peptide bond, resulting in conformational and functional changes of its substrate ( Liou et al. 2011 ; Cheng et al. 2013 ). It has been shown that PIN1 binds p27 and induces conformational change that hinders the binding between p27 and CKS1 ( Zhou et al. 2009 ). As a result, PIN1 positively regulates p27 level in competition with CKS1.

Apart from inhibiting cell-cycle entry, p27 is known to have cell-cycle independent roles in controlling neuronal migration in the developing mouse neocortex. In particular, stabilization of p27 by the neuronal-specific CDK, CDK5, has been shown to be crucial in this context ( Kawauchi et al. 2006 ). Studies on mitotic as well as neuronal cells have indicated that during migration, p27 mediates actin cytoskeleton reorganization through inhibition of RhoA, a small GTPase involved in the Rho/ROCK kinase signaling cascade ( Besson et al. 2004 ). An important downstream effector of this pathway is cofilin, which acts as an actin severer. LIM and other kinases have been shown to negatively regulate the activity of cofilin by phosphorylation of the serine residue at amino-acid position 3 ( Mizuno 2013 ). Precise control of cofilin activity is important in cellular migration as well as in processes involving actin cytoskeleton remodeling, such as dendritic spine maturation ( Pontrello and Ethell 2009 ; Tolias et al. 2011 ). Non-phosphorylated or activated cofilin promotes the formation of cofilin-actin rods or aggregates that are typically identified in neurons in neurodegenerative disorders such as Alzheimer's disease (AD) ( Huang et al. 2008 ).

In this study, we demonstrated that CKS1 possesses a cell-cycle independent role in controlling dendritic spine maturation. Through mediating p27 degradation, and thus RhoA activation, CKS1 negatively controls cofilin activity. CKS1 is expressed strongly in the adult hippocampus. The sequelae of *Cks1* deletion, hence cofilin over-activation and formation of cofilin-actin aggregates, include impaired maturation of hippocampal dendritic spines, an inability to establish late-phase long-term potentiation (LTP) and impaired hippocampal-based spatial learning.

## Materials and Methods

### Animals

All experiments were conducted in 3–4-month-old male *Cks1* knockout mice ( *Cks1*^−/−^ ) ( Frontini et al. 2012 ), and their wild type (WT) littermate controls. All procedures were conducted in accordance with the United Kingdom Animals (Scientific Procedures) Act, 1986 following institutional ethical approval at the University of Oxford and University of Sussex.

### X-Gal Histochemistry

Mice were anesthetized with chloroform and fix-perfused transcardially with 4% paraformaldehyde in 0.1 M sodium phosphate-buffered saline (PBS). Brains were removed and incubated in cold fix for an additional 15 min. After rinsing 3 times in rinse buffer (100 mM sodium phosphate (pH 7.3), 2 mM MgCl _2_ , 0.01% sodium deoxycholate, 0.02% Nonidet P40), sections were stained (rinse buffer plus 5 mM potassium ferrocyanide, 5 mM potassium ferricyanide, and 1 mg/ml 5-bromo-4-chloro-3-indolyl-beta-D-galactopyranoside) overnight at 4 °C. Sections were post-fixed before sectioning and mounting.

### Novel Object Recognition

The Novel Object Recognition (NOR) task was performed in an open-field apparatus (40 cm wide × 60 cm long × 40 cm high) placed under a dim light. In the habituation phase, mice were individually allowed to explore the box for 10 min per day for 3 days. In the training trial, mice were presented with 2 identical, emotionally neutral plastic objects (such as a red cube), placed in opposite corners of the box and allowed to explore for 5 min. In the test trial performed 24 h later, we replaced one of the familiar object with a new one (e.g. yellow pyramid). Objects were similar in size and emotionally neutral, but varied in shape and color. Mice were allowed to explore for 5 min and exploratory behavior recorded, i.e. head orientation, sniffing, nose to object distance ≤1 cm. All trials were video-tracked using Ethovision XT 7.1 (Noldus Information Technology). We determined a preference index (PI), which is a difference in the new object exploration time divided by the total time spent exploring the 2 objects. The open-field box and objects were thoroughly cleaned with 70% ethanol solution, dried, and ventilated between tested mice to eliminate odor cues.

### Barnes Maze Test

The Barnes maze consisted of a white, polypropylene disk of 100 cm diameter, mounted 50 cm above the floor. The disk has 20 holes of 5 cm diameter and evenly spaced around the periphery. A black acrylic drawer was located beneath one of the hole and served as the escape box. Fixed spatial cues were placed on the walls and a bright light (100 W) provided the aversive stimuli. The method was modified from Patil et al. (2009) . For each trial, mice were allowed to freely explore the maze for 2 min or until reaching the escape box, where they would remain for 1 min before being returned to their home cage. If a mouse failed to find the escape box within 2 min, the experimenter would gently direct the animal to the escape box where it would remain for 1 min. During 5 consecutive days mice had either 3 trials per day (with a retention time of 15 min) or 1 trial per day (retention time of 24 h). The time spent before finding the escape box was measured as an indication of spatial learning. All trials were video-tracked using Ethovision XT 7.1 (Noldus Information Technology), which recorded distance traveled and latency to find the escape box. The maze and escape box were cleaned carefully with a 70% ethanol solution between each trial to eliminate odor cues.

### Electrophysiology

Mice were anesthetized with pentobarbitone prior to decapitation and preparation of slices. Transversal hippocampal slices (350 μm) were cut with Microm HM650V slicer in ice-cold artificial cerebrospinal fluid (aCSF) comprising (in mM): 119 NaCl, 2.5 KCl, 2.5 CaCl _2_ , 1.3 MgSO _4_ , 1.25 NaH _2_ PO _4_ , 25 NaHCO _3_ , 11 glucose (pH 7.2–7.4, equilibrated with 95% O _2_ /5% CO _2_ ), stored at 20–24 °C for at least 1 h, then transferred to a submerged recording chamber perfused with aCSF at 32 °C for at least 30 min before recording. An extracellular concentric tungsten stimulation electrode was positioned in the CA1 *stratum radiatum* . A cut was made between the CA3 and CA1 areas to prevent polysynaptic activity. Single-shock stimuli were applied at 15 s interval, and field potential EPSPs (fEPSPs) were recorded in the CA1 *stratum radiatum* using glass capillary electrodes (5–10 MΩ) filled with aCSF. Stimulation intensity was set to 50% of maximal fEPSP amplitude. Theta-burst stimulation (TBS) comprised 10 trains of 5 pulses at 100 Hz separated by 200 ms. About 20–80% fEPSP slope was analyzed and magnitude of LTP was defined as % of baseline mean. Paired *t* -test was used to test significance. Miniature EPSCs (mEPSCs) were recorded in voltage clamp at −60 mV with Multiclamp 700B amplifier (Axon Instruments) and using filling solution (in mM): 135 CsCl, 10 KOH-HEPES, 10 BAPTA, 8 NaCl, 2 Mg-ATP, 0.3 GTP (pH 7.2, 290 mOsm/L). Neurobiotin (Vector Labs, UK) (0.3% w/v) was present for post hoc anatomical analysis. mEPSCs were analyzed from at least 5 min recording episodes, with stable access resistance (<25 MΩ). Data were low-pass filtered (4–5 kHz) and acquired at 10–20 kHz for off-line analysis. The GABA _A_ R blocker picrotoxin (100 μM) and tetrodotoxin (TTX) (1 μM) were present in all mEPSC experiments. Data were analyzed using pClamp 10 (Axon Instruments).

### Dendritic Spine Density and Structure Analysis

Slices were fixed with 4% PFA and 0.2% picric acid solution overnight at 4 °C, and then washed in 0.1 M phosphate buffer. Neurobiotin was visualized by incubating with Alexa Fluor 488-conjugated streptavidin (Invitrogen, UK; diluted 1:1000) in TBS with 0.3% Triton XC-100. Slices were mounted in Vectashield (Vector Laboratories). Fluorescent images of dendrites were collected using a confocal laser scanning microscope (Leica TCS SP5). At least 3 randomly selected areas with 50–70 μm length each were imaged from a single pyramidal cell. About 3 or more cells were analyzed from each animal and having at least 3 animals per genotype. Dendritic spines morphology was analyzed using the NeuronStudio software ( Rodriguez et al. 2008 ).

### siRNA Transfection

Specific siRNA targeting Cks1 (si *Cks1* ) and control siRNA (siCrtl) were purchased from Dharmacon (SMARTpools) and the cells were transfected with Oligofectamine transfection reagent (Life-Technologies-Invitrogen) following the manufacturer's instructions. Cells were analyzed 48 h after transfection.

### Real-Time PCR and Gene Expression Analysis

mRNA of the hippocampus tissues was extracted by TRIzol reagent (Life Technologies) and cDNA was reverse transcribed using SuperScript III Reverse Transcriptase (Life Technologies). Power SYBR Green PCR Master Mix (Applied Biosystems, Life Technologies) was used for Real-time PCR. Transcript expression is presented as average Ct. Primer sequences for *Cks1* mRNA are TACGACGACGAGGAGTTCGAAT (Forward) and ACCAGCTTGGCTATGTCCTTGGG (Reverse). The fold changes for *Cks1* transcript from hippocampus of WT mice relative to that of *Cks1*^−/−^ mice was calculated with the 2 ^−ΔΔCt^ method ( Huggett et al. 2005 ).

### Immunoprecipitation and Western Blotting

Hippocampal extracts were made from homogenized *Cks1*^−/−^ and WT brains in lysis buffer containing 5 mM HEPES (pH 7.3), 200 mM NaCl, 1.5 mM MgCl _2_ , 0.2 mM EDTA, 20 mM β-glycerol phosphate, 1 mM sodium orthovanadate, 0.5% Triton X-100, and 5% glycerol with protease inhibitors (Roche). Primary hippocampal neurons were made from E14 embryos (see below). Anti-p27 antibody (#sc-528, Santa Cruz Biotechnology) was firstly cross-linked to Protein A/G agarose beads (Thermofisher) using dimethyl pimelimidate dihydrochloride (Sigma). Extracts were incubated with antibody-conjugated beads at 4 °C overnight. After extensive washing, the immunoprecipitates were separated by SDS-PAGE for Western blot analysis with anti-p27 antibody (#610241, BD Transduction Laboratories) and anti-RhoA antibody (#sc-418, Santa Cruz Biotechnology). For active RhoA pull-down, a GSTRhotekin-RBD column was used according to the manufacturer's protocol (#16116, Thermofisher). Cofilin was detected using the following antibodies: anti-cofilin (#5175, Cell Signaling), anti-Ser3 cofilin (#3313, Cell Signaling).

### Culture of Primary Hippocampal Neurons and Immunostaining

The primary hippocampal neurons were derived from the E14 embryos of WT and *Cks1*^−/−^ mice. The neurons were seeded on 24-well plates with coverslips coated with poly-L-lysine with Neurobasal Medium (Life Technologies) and B27 supplement (50×). Immunostaining was then performed on the primary neurons after culture for 14 days. For neurons immunostaining, cells were rinsed with PBS twice and fixed for 30 min in a solution of 4% paraformaldehyde, pH 7.4. Coverslips were then rinsed 3 times in PBS and permeablized with ice-cold methanol for 90 s. Permeablization solution was removed and washed 3 times with PBS. Anti-cofilin antibody (#5175, Cell Signaling) and anti-beta III tubulin antibody [TUJ-1] (ab14545, Abcam) were added together with a donkey serum blocking solution in PBS and set to incubate overnight at 4 °C. Coverslips were then rinsed 3 times in PBS for 10 min. Samples were further incubated for 1 h in anti-rabbit Alexa Fluor 488-conjugated secondary antibodies and anti-mouse Alexa Fluor 647-conjugated secondary antibodies (Invitrogen). The slips were washed finally with PBS for 3 times. Images were taken with Carl Zeiss LSM 510 Meta/Axiocam. Cofilin aggregations were counted with ImageJ software with puncta analyzer plug-in (National Institutes of Health). Details of the quantification method using this plug-in have been described previously, and each of the image backgrounds was subtracted (rolling ball radius = 50) in order to detect discrete puncta (cofilin aggregates) without introducing background noise ( Ippolito and Eroglu 2010 ).

### Statistical Tests and Analyses

Significance was analyzed either with the Mann–Whitney test or *t* -test, and for the multiple parameter comparisons with 1-way ANOVA and post hoc Bonferroni or Tukey's test. Parametric distribution of data was tested with Shapiro–Wilk test.

## Results

### CKS1 is Involved in Establishment of Long-Term Memory in Adult Hippocampus

**Figure 1.**
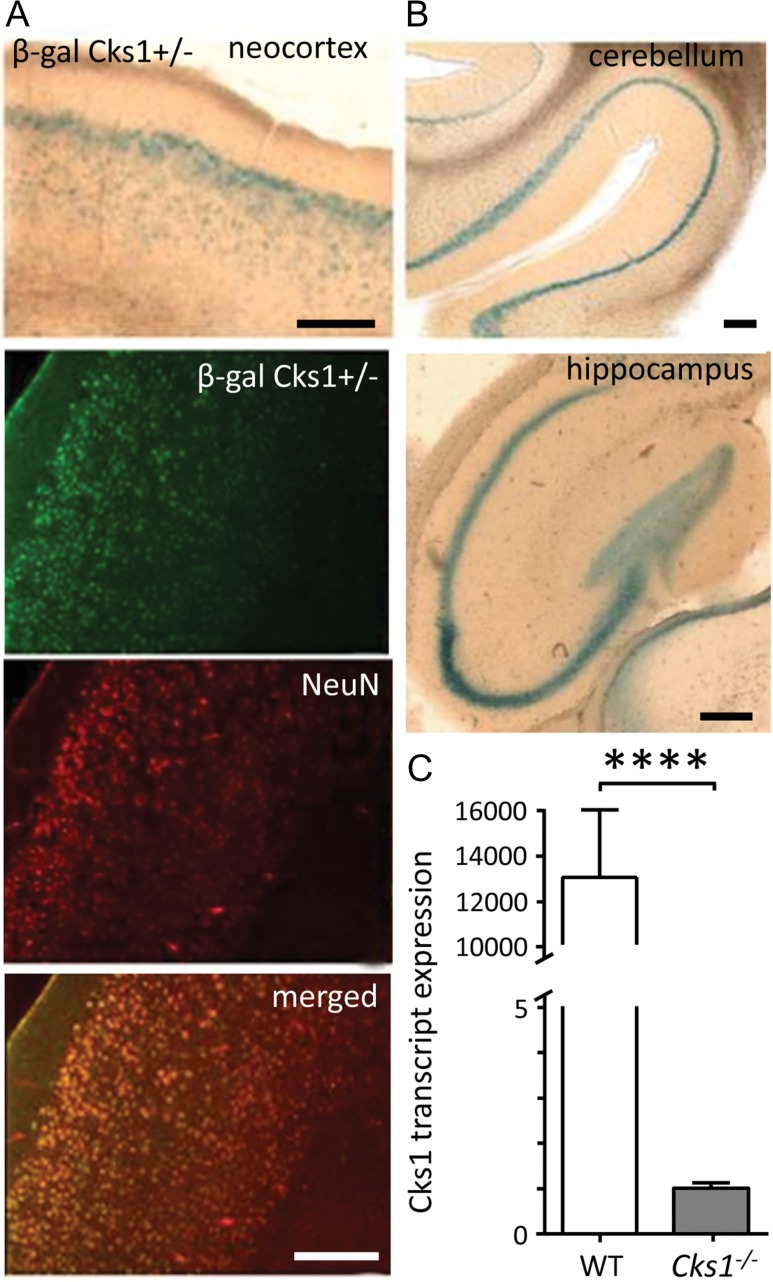
CKS1 is expressed in the adult mouse brain including hippocampus. ( *A* ) In mice heterozygous for the *Cks1* knockout cassette that harbors a LacZ insertional cassette for gene disruption ( *Cks1*^*+/−*^ ), β-galactosidase activity acts as a marker for *Cks1* gene expression. Above: Prominent *Cks1* expression in the entorhinal cortex, particularly in the layer 2–3 (coronals section of a 4-month-old male). Below: Confocal fluorescence images showed that neurons expressing *Cks1* were mature neurons as they also expressed the neuronal marker NeuN. Coronal section stained with an anti-β-galactosidase antibody (β-gal Cks1 ^+/−^ ), anti-NeuN antibody (NeuN), and image overlap (merged). Scale 200 μm. ( *B* ) The β-galactosidase activity was detected in various brain areas including the cerebellum showing staining in Purkinje cells and the hippocampal CA1–CA3 area and the dentate gyrus. Scale 200 μm. ( *C* ) Real-time PCR of *Cks1* transcript in hippocampus of WT and *Cks1*^−/−^ mice. The average Ct obtained in *Cks1*^−/−^ mice was undetermined and was set as 40 for calculation. The fold change (2 ^-ΔΔCt^ ) was calculated relative to *Cks1*^−/−^ ( *P* <0.0001, *t* -test). Data ( *n* = 5) were expressed as mean ± SEM. *GAPHD* was used as internal control.

During investigation of CKS1 in the developing murine cortex, we recently observed that apart from controlling cell-cycle exit during neurogenesis, CKS1 was also actively expressed in mature neurons ( Frontini et al. 2012 ). We therefore examined *Cks1* expression in the adult brain by using β-galactosidase activity as a marker in heterozygous mice where one copy of *Cks1* was disrupted by a *LacZ* insertion cassette. *Cks1* expression was detected in various regions of the brain (Fig. 1*A* ), including the hippocampus (Fig. 1*B* ). Adult hippocampal expression of *Cks1* was further confirmed with quantitative real-time PCR (RT-PCR) of RNA extracted from the hippocampi of WT mice (Fig. 1*C* ). Given the well-established role of CKS1 in dividing cells, we expected to see *Cks1* expression in areas where adult neurogenesis occurs ( Drew et al. 2013 ), but not in post-mitotic neurons. Surprisingly, in addition to the dentate gyrus, *Cks1* expression was detected in the CA1, CA2 and CA3 areas and particularly in the *stratum pyramidale* .

To determine whether the *Cks1* expression has significance in brain function, we compared the behavior of *Cks1* knockout ( *Cks1*^−/−^ ) mice with WT littermates. Although *Cks1*^−/−^ mice were physically smaller than WT mice, possibly related to the accumulation of p27 ( Spruck et al. 2001 ), they did not show statistically different performance on the standardized SHIRPA protocol ( Supplementary Table S1 ), and no motor deficits were observed.

**Figure 2.**
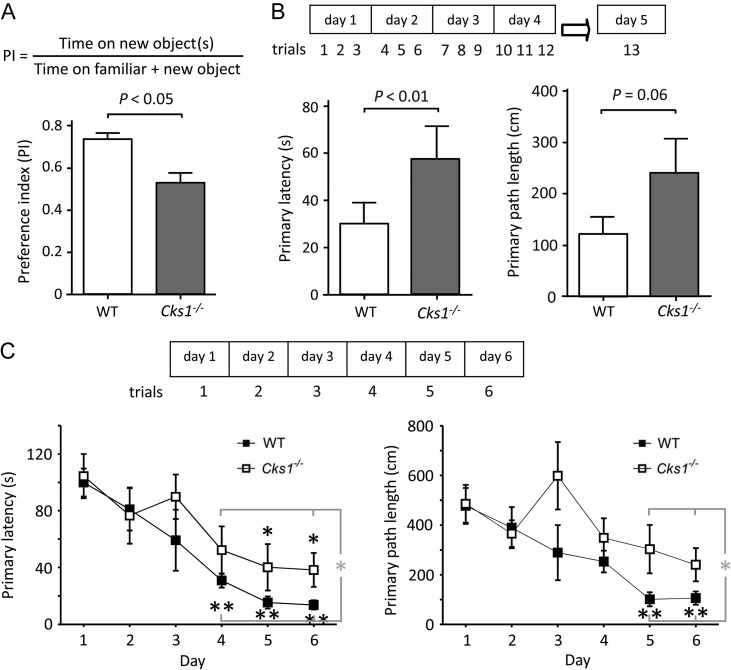
*CKS1* is required for hippocampus-dependent learning and long-term memory. ( *A* ) *Cks1*^*−/−*^ mice were deficient in NOR. Average novel object PI (mean ± SD) in at least 4 animals of each genotype (* *P* <0.05, *t* -test). ( *B,C* ) *Cks1*^*−/−*^ showed impaired learning in the Barnes maze test. ( *B* ) WT and *Cks1*^*−/−*^ males were trained and assessed with a retention time of 15 min, having 3 trials per day for 4 days (trials 1–12) and tested on the fifth day to study whether they remembered the route to the escape hole without further training, as shown in a scheme on top. Animals were individually video-tracked using the EthoVision system. Left: Time (mean ± SD) taken for an animal to reach the escape hole on day 5 was plotted as primary latency ( *n* = 18 in each group, ** *P* <0.017, *t* -test). Right: The total distance traveled as primary path length ( *n* = 18 in each group, *P* = 0.05, *t* -test). ( *C* ) *Cks1*^*−/−*^ animals also showed a defect in memory on a training protocol as in ( *B* ), but having trials with 24 h intervals for 6 days (scheme on top). Left: WT mice showed significant change in the primary latency from the training day 4 onwards (compared with day 1, black asterisks ** *P* <0.01, 1-way ANOVA with Bonferroni test), whereas *Cks1*^*−/−*^ mice showed difference from day 5 onwards (black asterisk * *P* <0.05). The genotypes were significantly different at days 4–6 (gray asterisk * *P* <0.05). Right: Correspondingly, the WT showed significant change in primary path length from day 5 onwards (compared with day 1, black asterisks ** *P* <0.01, 1-way ANOVA with Bonferroni test) whereas in the *Cks1*^*−/−*^ mouse population this was not significantly changed from day 1 in any of the days. The days 5–6 results were significantly different between the WT and the *Cks1*^*−/−*^ mice (gray asterisk * *P* <0.05) ( *n* = 6 in each group). In addition, analysis of the pooled data (from days 1 to 6) by repeated measure ANOVAR showed a statistical significance between WT and *Cks1*^*−/−*^ animals: primary path length, *P* <0.026; primary latency, *P* <0.045.

Given the prominent CKS1 expression in the adult hippocampus, we tested *Cks1*^−/−^ mice and their WT littermate controls in a NOR task. *Cks1*^−/−^ mice displayed a statistically significant ( *P* <0.05) decrease in preference for a novel object (Fig. 2*A* ). To further investigate if this was due to a hippocampal defect, hippocampus-dependent spatial learning and memory were examined using Barnes circular maze ( Patil et al. 2009 ). Performance was measured by the time (primary latency) and distance (primary path length) taken for an animal to reach an escape hole from the open surface of a Barnes maze arena. Mice were initially given 3 learning trials, 15 min apart every day for 4 days. After 12 test trials, animals were tested on the fifth day to see whether they remembered the route to the escape hole without further training. If acquisition of new memory was normal, *Cks1*^−/−^ mice and their WT control animals would be expected to demonstrate a similar degree of reduction in latency. However, the *Cks1*^−/−^ mice spent a significantly longer time ( *P* <0.01, *t* -test) and used a longer path, very close level of significant difference the WT ( *P* = 0.05), to reach the escape hole, reflecting a defect in establishing long-lasting memory ( *n* = 18 mice in both groups) (Fig. 2*B* ). We next performed the Barnes maze experiment on a separate cohort of mice, but providing the mice 1 trial per day for 6 days (i.e. a retention time of 24 h) instead of 3 temporarily closely spaced trials each day (i.e. a retention time of 15 min each). Similarly, *Cks1*^−/−^ mice exhibited a significant impairment in memory as reflected by the longer primary latency and longer primary path length taken by them to reach the escape hole on days 4–6 and 5–6, respectively ( *P* <0.05 for both, *t* -test), compared with WT littermates (Fig. 2*C* ). To support the contention that WT mice and *Cks1* -/- mutants are distinct in their learning ability and not their performance in the Barnes maze per se, we tested an independent group of mice in the Barnes maze. Our results confirmed that WT mice ( *n* = 6) and *Cks1−/−* mutants ( *n* = 6) have no performance difference the first time they encounter the maze (primary latency, *P* = 0.64; primary path length *P* = 0.70). Collectively, our results highly suggested that CKS1 in the adult hippocampus is required for normal acquisition and consolidation of memory.

### CKS1 is Required for Late LTP and Dendritic Spine Maturation

**Figure 3.**
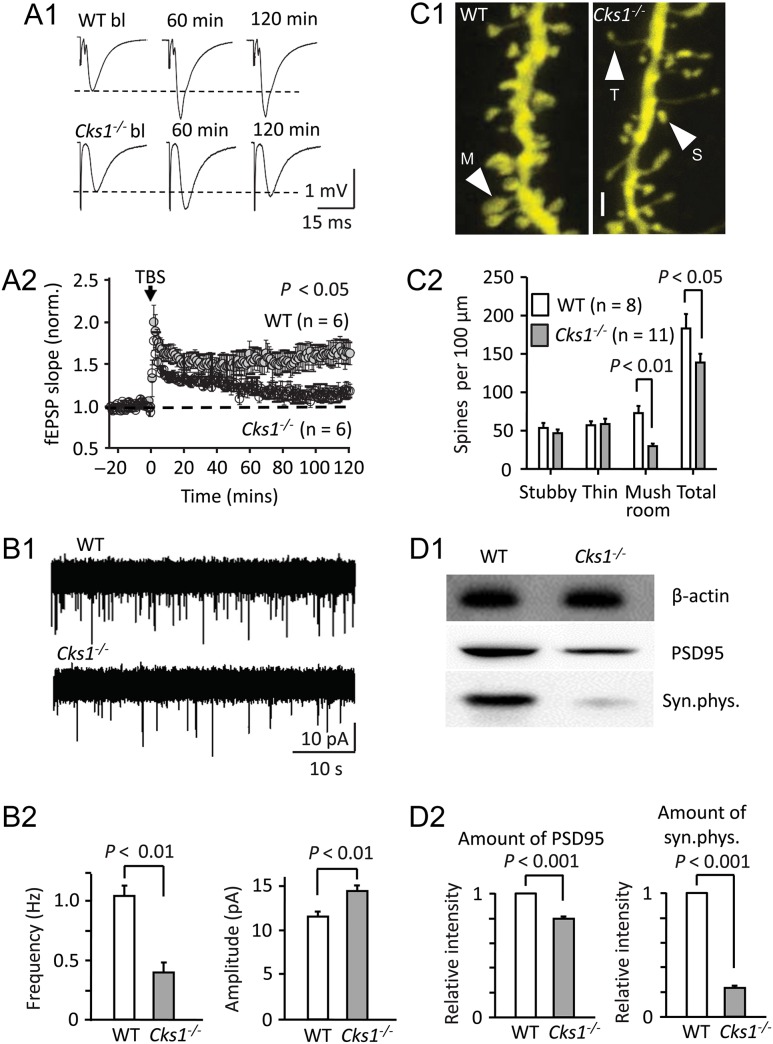
Impaired LTP and dendritic spine maturation in *Cks1*^*−/−*^ mice. ( *A* ) *Cks1*^*−/−*^ mice exhibited impaired late-phase LTP. ( *A1* ) Averaged field potential EPSP (fEPSP) from a sample experiment in WT and in *Cks1*^*−/−*^ mouse during baseline (bl), and at a time point of early (60 min) and late LTP (120 min) elicited by TBS. ( *A2* ) Mean ± SEM of fEPSP slope (baseline-normalized) in the hippocampal Schaffer collateral—CA1 pathway in WT and *Cks1*^*−/−*^ mice. Arrow indicate LTP induction with TBS (WT, gray symbols; *Cks1*^*−/−*^ , open symbols). In both genotypes early phase LTP (up to 60 min) was observed ( *t* -test). Yet, slices from *Cks1*^*−/−*^ mice failed to establish late-phase LTP (from 60 to 120 min) ( *n* = 6 in both groups, *t* -test). *P* <0.05 between the groups at 60–120 min ( *t* -test). ( *B* ) Pyramidal cells in *Cks1*^*−/−*^ mice showed decreased frequency of quantal miniature EPSCs (mEPSCs). ( *B1* ) Sample traces from individual recordings showing glutamatergic mEPSCs in CA1 area pyramidal cells (at −60 mV) in a WT and a *Cks1*^*−/−*^ mouse hippocampal slice. ( *B2* ) Mean ± SEM of mEPSC frequency (left) and amplitude (right) in all studied cells. The mEPSC frequency was robustly decreased in *Cks1*^*−/−*^ cells ( *t* -test). In addition, mEPSC amplitude showed moderate increase ( *t* -test). ( *C* ) Decreased mushroom spine density in *Cks1*^*−/−*^ pyramidal cells. ( *C1* ) Confocal images of CA1 area pyramidal cell dendrites showing the 3 spine types in the WT and *Cks1*^*−/−*^ mice. Characteristic ‘thin’ (T), ‘stubby’ (S), and ‘mushroom’ (M) spines were indicated by arrowheads. Scale 1 μm. Semi-automated scoring of the spine types was carried out using the NeuronStudio software. ( *C2* ) Mean ± SEM of the density of the spine types ( *t* -test). ( *D* ) Levels of PSD95 and synaptophysin were lower in primary hippocampal neurons derived from *Cks1*^*−/−*^ mice than WT controls. ( *D* 1) Band intensities in Western blot experiments were normalized using β-actin as loading control. ( *D2* ) Histograms showing relative intensity of PSD95 (left) and synaptophysin (right) in WT controls and *Cks1*^*−/−*^ mice neurons. Data were expressed as mean ± SEM ( *n* = 5).

We suspected *Cks1*^−/−^ mice would have difficulties to establish LTP, which is considered to be the cellular substrate of hippocampal memory formation ( Bliss and Collingridge 1993 ). Studying field excitatory postsynaptic potential (fEPSP) in acute hippocampal slices, we observed that both WT and *Cks1*^−/−^ mice establishing early (<60 min from TBS, post-TBS) LTP (Fig. 3*A1* , *A2* ). The recordings were made with standard extracellular solution without added drugs. At 60 min post-TBS, the fEPSP potentiation was significant from baseline (15 min) in the WT mice hippocampal Schaffer collateral pathway (1.38 ± 0.08, *n* = 14, *P* <0.01) as well as in the *Cks1*^−/−^ (1.26 ± 0.05, *n* = 15, *P* <0.01) (at 50–60 min from TBS). Although average early LTP was moderately smaller in the *Cks1*^−/−^ mice, there was significant difference between the 2 genotypes ( *P* = 0.18, *t* -test). In recordings following the fEPSP for 2 h post-TBS, we found that the late LTP was compromised in *Cks1*^−/−^ mice (1.10 ± 0.03, *n* = 6) as compared with the littermate controls (1.65 ± 0.14, *n* = 6) (Fig. 3*A2* ) ( *P* <0.01 comparing baseline-normalized fEPSPs between the groups at 110–120 min post-TBS, *n* = 6 and 6, *t* -test). Thus, the inability of *Cks1*^−/−^ mice to establish late LTP may at least partially explain the memory defects we observed on the NOR experiment and the Barnes maze.

Late LTP requires protein synthesis and consolidation of synaptic plasticity in postsynaptic sites ( Bramham 2008 ). LTP establishment also involves growth of dendritic spines, the actin-based membrane protrusions where the majority of excitatory synapses reside. To investigate whether the density of excitatory synapses in CA1 pyramidal cells were altered, we recorded glutamatergic miniature EPSCs (mEPSCs) generated by stochastic release of synaptic transmitter vesicles in the presence of tetrodotoxin, 1 μM and the GABA _A_ R blocker, picrotoxin, 100 μM (Fig. 3*B1* , *B2* ). Pyramidal cells in *Cks1*^−/−^ mice showed significantly lower mEPSC frequency (0.39 Hz, *n* = 12) than those of WT littermates (1.04 Hz, *n* = 11) ( *P* <0.01, *t* -test) (Fig. 3*B2* ). In addition, we found that mEPSC amplitudes were moderately higher in the *Cks1*^−/−^ mice (13. 8 ± 0.5 pA) than in control cells (11.5 ± 0.6 pA, *P* <0.01, *t* -test). These findings suggest that hippocampal CA1 pyramidal cells in the *Cks1*^−/−^ mice have lowered density of afferent glutamatergic synapses and the average strength of a quantal current is moderately stronger than in the WT hippocampus.

During learning, neuronal activity is known to facilitate the growth of dendritic spines that results in an increase in spine head area-to-length ratio (hence formation of the so-called mushroom spines from filopodia or thin spines). This type of structural plasticity is commonly associated with establishment of late LTP ( Bosch and Hayashi 2012 ). Hence, we studied whether dendritic spine maturation differed between *Cks1*^−/−^ and WT mice. We visualized and analyzed dendrites of pyramidal cells (which we previously made electrophysiological recordings from and filled them with neurobiotin) in the hippocampal CA1 area with confocal microscopy. We found that *Cks1*^−/−^ pyramidal cell apical dendrites were deficient in mushroom spines, which represent the mature spine form (Fig. 3*C1* ). The relative proportion of mushroom spines in dendrites was significantly lower in *Cks1*^−/−^ pyramidal cells (21 ± 4%, *n* = 8, *P* <0.01) than in WT pyramidal cells (38.6 ± 6%) (Fig. 3*C2* ). Moreover, to assess the presence of dendritic synapses, we examined the expression levels of synaptophysin and PSD95 (pre-synaptic and postsynaptic markers, respectively) in the primary hippocampal neurons from E14 embryo of the *Cks1*^−/−^ and the WT mice using immunoblotting. As shown in Figure 3*D1* , *D2* , the expression levels of synaptophysin and PSD95 were significantly lower in the *Cks1*^−/−^ neurons, suggesting that fewer synapses were present in the *Cks1* null neuronal dendrites. All these results supported the notion that CKS1 contributes to formation of dendritic spines and in its absence, the establishment of late LTP and long-term memory, development of mushroom-shape spines and functional dendritic synapses are compromised.

### CKS1 Controls Phosphorylation of Cofilin Through Destabilization of p27 and Activation of RhoA Kinase

**Figure 4.**
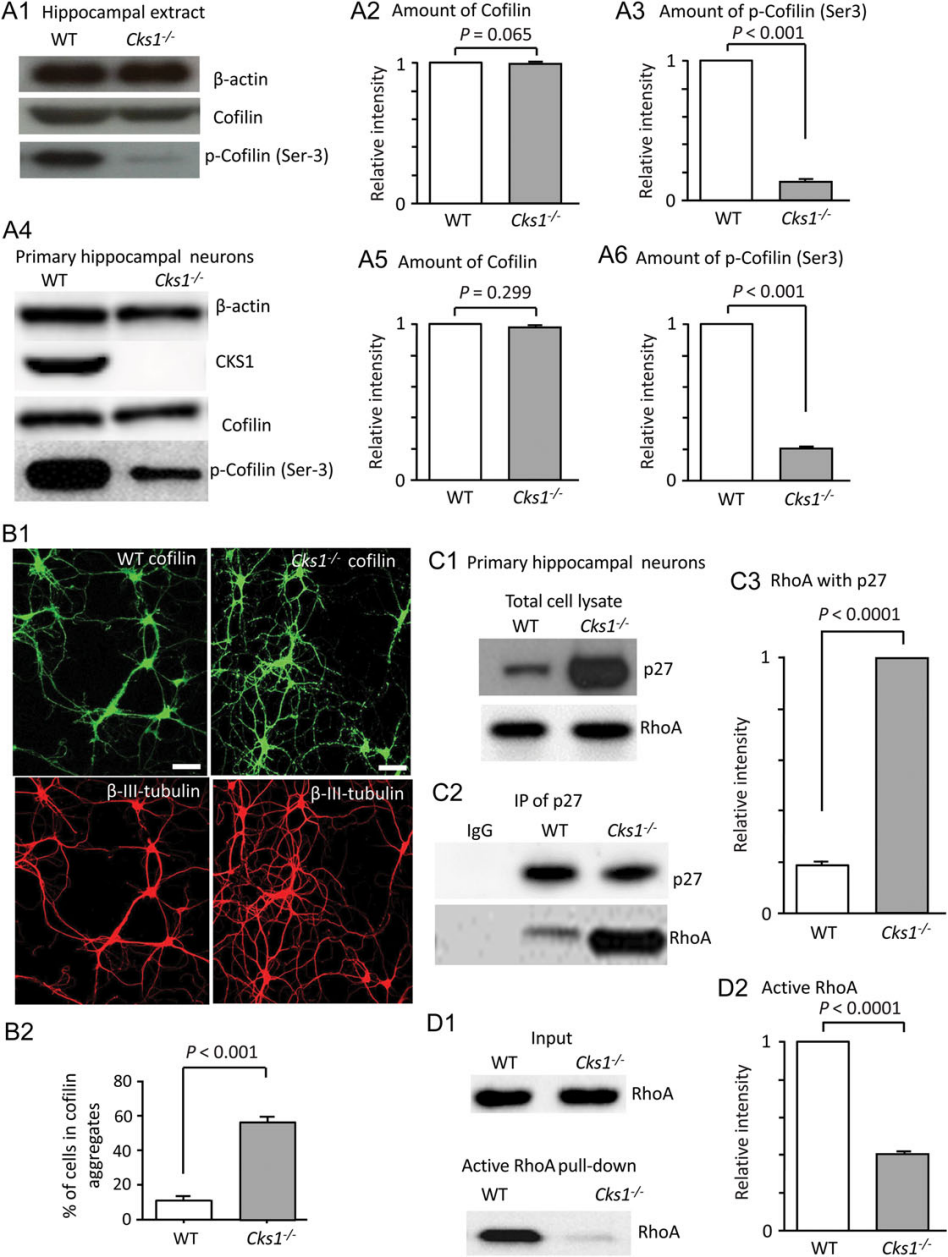
CKS1 controls the phosphorylation of cofilin at Ser3 site and the formation of cofilin aggregates by limiting binding of p27 to RhoA in primary hippocampal neurons. ( *A* ) The level of cofilin phosphorylated at Ser3 both in *Cks1*^*-/-*^ hippocampal extracts and in primary hippocampal cells was lower than that in the WT hippocampal controls. ( *A1* ) Western blot of hippocampal extract using β-actin as loading control. ( *A2* ) Histogram showing relative intensity of total cofilin in hippocampal extracts. Data were expressed as mean ± SEM ( *n* = 9). ( *A3* ) Histogram showing relative amount of p-cofilin (Ser3) in hippocampal extract. Data were expressed as mean ± SEM ( *n* = 9). ( *A4* ) Western blot of primary hippocampal neurons with β-actin as loading control. ( *A5* ) Relative intensity of total cofilin in primary hippocampal neurons. Data were expressed as mean ± SEM ( *n* = 9). ( *A6* ) Relative amount of p-cofilin (Ser3) in primary hippocampal neurons. Data were expressed as mean ± SEM ( *n* = 9). ( *B* ) The percentage of primary hippocampal neurons with aggregates was significantly higher in the *Cks1*^*-/-*^ genotype than in WT derived neurons. ( *B1* ) Confocal immunofluorescence images illustrate aggregates in WT and *Cks1*^*-/-*^ neurons. Scale 50 μm. ( *B2* ) Histogram showing % of cells associated with the aggregates. ( *C* ) Increased amount of RhoA bound to p27 in *Cks1*^*-/-*^ cells; ( *C1,C2* ) Immunoprecipitation of p27 showed that more RhoA was bound to p27 in *Cks1*^*-/-*^ primary hippocampal neurons than in the WT. ( *C3* ) Histogram showing amount of RhoA interacting with p27 in primary hippocampal neurons. Data were expressed as mean ± SEM ( *n* = 9). ( *D* ) A GST-rhotekin column was used to pull-down active RhoA in WT or *Cks1*^*-/-*^ primary hippocampal neurons. ( *D1* ) By using antibody specific to RhoA, more active RhoA was detected in WT primary hippocampal neurons than in *Cks1*^*-/-*^ mice cells. Input showed the same amount of total RhoA used in the RhoA pull-down assay for WT and *Cks1*^*-/-*^ . ( *D2* ) Histograms showing the amount of active RhoA in primary hippocampal neurons. Data were expressed as mean ± SEM ( *n* = 9).

Dendritic spine maturation requires active actin cytoskeleton remodeling ( Calabrese et al. 2006 ). Cofilin, an actin cytoskeleton severer, is essential in this process. Previous studies have shown that expression of a constitutively active non-phosphorylatable cofilin inhibited dendritic spine maturation ( Shi et al. 2009 ). To examine whether the inactive phosphor-Ser3 form of cofilin in hippocampal extract of *Cks1*^−/−^ mice was reduced, an antibody that specifically recognizes the phosphor-Ser3 form of cofilin was used. Immunoblotting showed that phospho-Ser3 cofilin was significantly reduced in hippocampus of *Cks1*^−/−^ mice (Fig. 4*A1* – *A3* ). Similarly, the level of phospho-Ser3 cofilin was also markedly lower in *Cks1*^−/−^ primary hippocampal neurons than that of the WT (Fig. 4*A4* – *A6* ). Because previous studies have shown that non-phosphorylated cofilin aggregation induces synaptic loss in hippocampal neurons ( Cichon et al. 2012 ), we investigated if there was also increased cofilin aggregation in *Cks1*^−/−^ primary hippocampal neurons (Fig. 4*B1* ). The percentage of *Cks1*^−/−^ primary hippocampal neurons with cofilin aggregates (59.47 ± 2.58%, *n* > 200) was significantly higher ( *P* <0.0001) than that in WT neurons (11.33 ± 2.25%, *n* > 200) (Fig. 4*B2* ). Neuron-specific class III β-tubulin, a neural specific marker, was used to outline the normal neuronal morphology.

**Figure 5.**
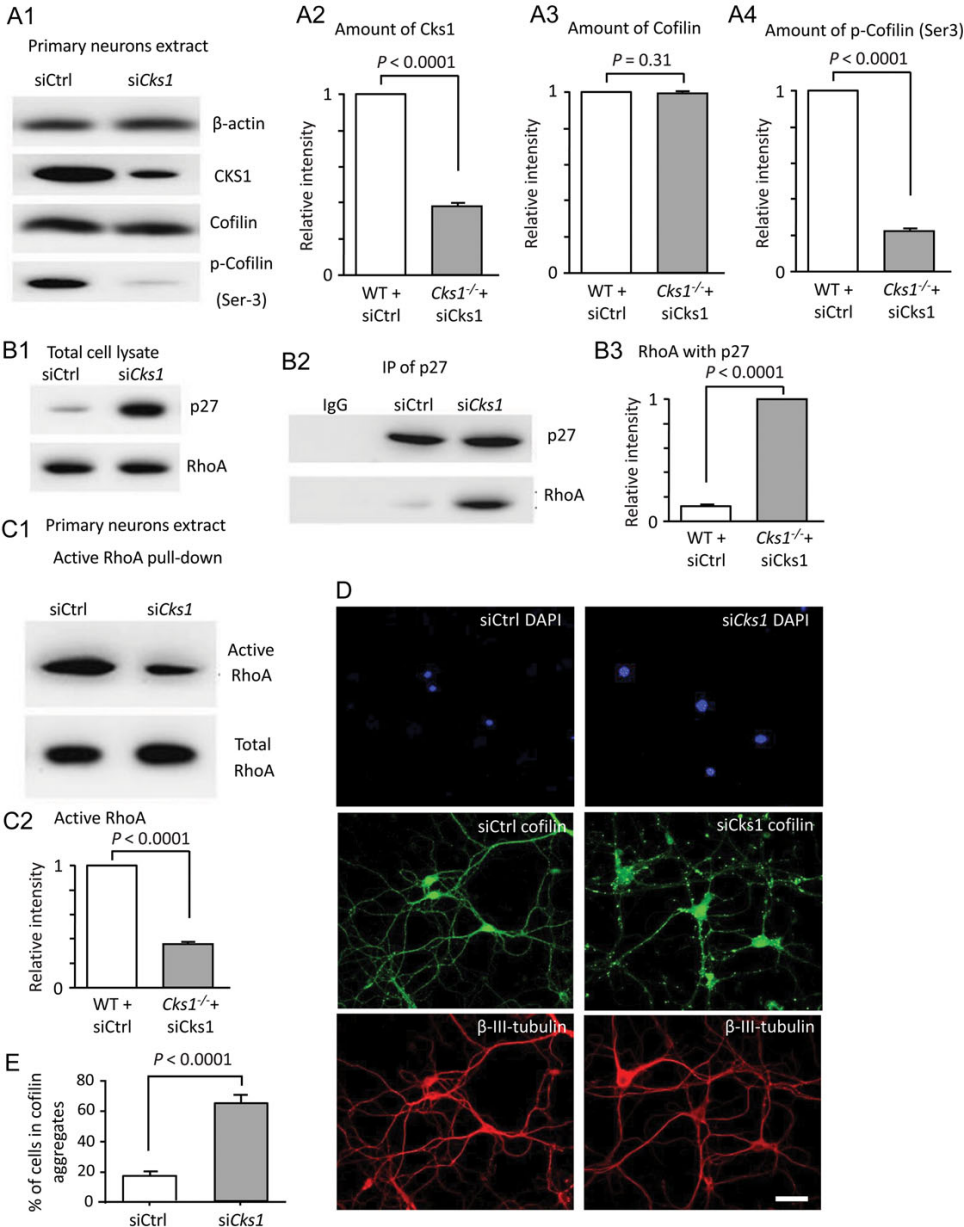
*Cks1* knocked down in WT primary hippocampal neurons leads to increased cofilin aggregates. WT primary hippocampal neurons were treated with either scrambled control siRNA (siCrtl) or siRNA against *Cks1* (si *Cks1* ). The effects of CKS1 on p27, RhoA and cofilin were examined with immunoblotting. The results of these experiments were similar to the results observed in *Cks1*^*-/-*^ primary hippocampal neurons. ( *A* ) The expression of CKS1 in WT primary hippocampal neurons was down-regulated by si *Cks1* , resulting in decreased p-cofilin. ( *A1* ) Western blot of using β-actin as loading control. ( *A2* – *A4* ) Histograms showing relative intensity of CKS1, cofilin, and p-cofilin (Ser3) in primary neuron extracts. Data were expressed as mean ± SEM ( *n* = 9). ( *B* ) Immunoprecipitation of p27 showed that more RhoA was bound to p27 in the cells treated with si *Cks1* . ( *B1* , *B2* ) Western blot with β-actin as loading control. ( *B3* ) Histograms showing amount of RhoA interacting with p27 in primary neuron extract. Data were expressed as mean ± SEM ( *n* = 9). ( *C* ) From active RhoA pull-down, more active RhoA was present in the primary hippocampal neurons treated with siCrtl. ( *C1* ) Western blot showing active and total RhoA. ( *C2* ) Histograms showing relative intensity of active RhoA in primary neuron extracts. Data were expressed as mean ± SEM ( *n* = 9). ( *D* ) WT primary hippocampal neurons treated with siCtrl or si *Cks1* . Confocal fluorescence micrographs showing DAPI and immunohistochemical reaction against cofilin and tubulin. Scale 50 μm. ( *E* ) The percentage of cofilin aggregates was significantly higher in the neurons treated with si *Cks1* than siCtrl ( *t* -test). Scale 50 μm.

Ser3 phosphorylation of cofilin is mediated by GTPase RhoA, which is in turn negatively regulated by p27 ( Kawauchi et al. 2006 ; Belletti et al. 2010 ). Given that *Cks1*^−/−^ mice accumulate high levels of p27, we hypothesized that RhoA was inhibited due to increased RhoA bound to p27, resulting in decreased cofilin phosphorylation in the brain of *Cks1*^−/−^ mice. To test this, we examined RhoA binding to p27 in primary hippocampal neurons of WT and *Cks1*^−/−^ mice. Immunoprecipitation experiments showed increased amount of RhoA bound to p27 in the *Cks1*^−/−^ background (Fig. 4*C1* – *C3* ). To confirm if the increased binding to p27 resulted in suppression of RhoA activity in *Cks1*^−/−^ mice, we employed a Rhotekin Rho binding-domain column to enrich for active GTP-bound Rho kinases. We found that *Cks1*^−/−^ primary hippocampal neurons indeed harbored less active RhoA than the WT littermate controls (Fig. 4*D1* , *D2* ). To further validate the effect of CKS1 on p27-RhoA axis, *Cks1* was knocked down with siRNA in primary hippocampal neurons from WT mice (Fig. 5 ). Consistent with the findings in *Cks1*^−/−^ mice, knocking down *Cks1* in WT neurons resulted in reduced amount of CKS1 (Fig. 5*A* ), increased p27 level and p27-RhoA binding (Fig. 5*B1* – *B3* ), inhibition of RhoA (Fig. 5*C1* , *C2* ), and increase in the formation of cofilin aggregates (Fig. 5*D* , *E* ). Taken together, these findings suggested that CKS1 is required for Ser3 phosphorylation of cofilin and for preventing cofilin aggregates formation.

### Decreasing p27 Level by PIN1 Inactivation Reduces Cofilin Aggregate Formation in *Cks1*^−/−^ Hippocampal Neurons

Cofilin aggregation and rod-like aggregate formation are associated with the development of neurodegenerative diseases, such as AD ( Bamburg et al. 2010 ). The rods have been shown to be co-localized with phosphorylated tau and responsible for phosphorylated tau accumulation in striated neuropil threads ( Whiteman et al. 2009 , 2011 ), a characteristic of tau pathology in the early stage of AD brain. The peptidyl-prolyl-isomerase PIN1 has been shown to compete with CKS1 for interaction with p27 and suppression of PIN1 activity is associated with destabilization of p27 ( Zhou et al. 2009 ). As shown in Figure 6*A* , the cofilin aggregates gradually disappeared with treatment of increasing concentration of PiB, an inhibitor of PIN1, in primary hippocampal neurons from *Cks1*^−/−^ mice. PiB treatment reduced the p27 level, decreased the interaction between p27 and RhoA, and increased phosphorylation of cofilin (Fig. 6*B1* – *B7* ). Similarly, in WT primary hippocampal neurons with *Cks1* knocked down by siRNA, PiB treatment also resulted in diminished number of cofilin aggregates (Fig. 6*C* ). In addition, it decreased binding between p27 and RhoA, and increased phosphorylation of cofilin (Fig. 6*D1* – *D7* ). Treatment with PINTIDE, a very specific PIN1 inhibitory phosphopeptide, also showed similar biochemical results, confirming the effect of PIN1 inhibition in reversing the cofilin aggregates formation (Fig. 7*A* – *C* ). Taken together, the results suggested that regulation of p27 level via PIN1 and CKS1 determines the activity of cofilin and the formation of cofilin aggregates.

**Figure 6a.**
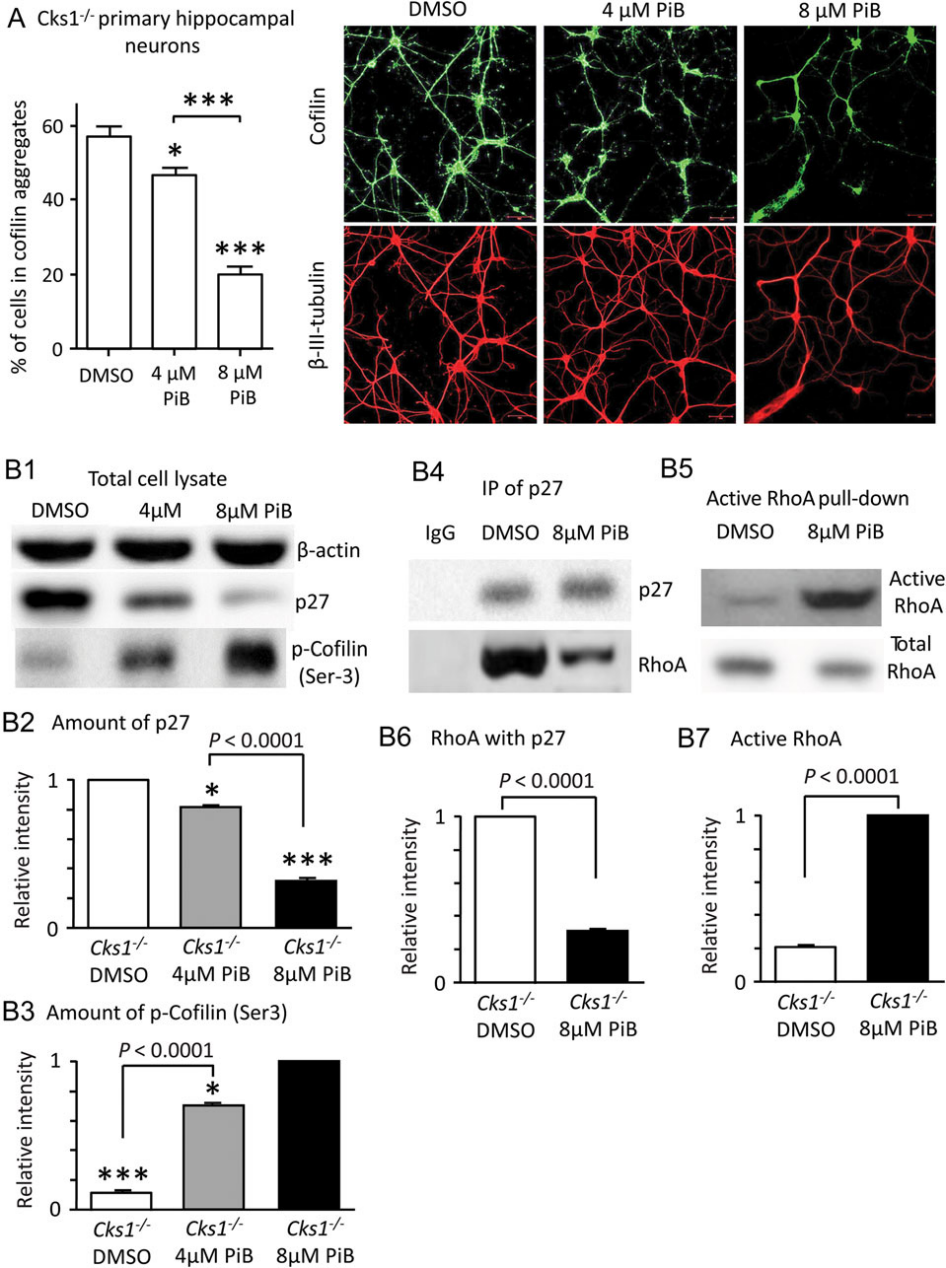
Inhibition of PIN1 diminished cofilin aggregates through lowering p27 levels. ( *A* ) PiB treatment effectively reduced cofilin aggregates in the *Cks1*^*-/-*^ primary hippocampal neurons in a dose dependent manner compared to control (DMSO) (* *P* <0.05, *** *P* <0.001, 1-way ANOVA with Tukey's test). Confocal images showing cofilin and tubulin staining in the different conditions. Scale 50 μm. ( *B* ) In *Cks1*^*-/-*^ primary hippocampal neurons, PiB treatment decreased p27 level and increased phosphorylation of cofilin via controlling the activity of RhoA. ( *B1* ) Western blot showing p27 and p-cofilin (Ser3) levels in primary hippocampal neuron extract. ( *B2* ) Histogram showing relative intensity of p27 compared to control (DMSO) (* *P* <0.05, *** *P* <0.001, *t* -test). There is highly significant difference between PiB treatment with 4 μM and 8 μM PiB (1-way ANOVA with Tukey's test) ( *n* = 9). ( *B3* ) Histogram shows relative intensity of p-cofilin (Ser) ( *n* = 9). ( *B4* ) This was associated with less RhoA binding with p27 (Western blot). ( *B5* ) More active RhoA was observed in PiB treated cells (Western blot). ( *B6* ) Histogram showing the relative (compared to control treatment with DMSO) amount of RhoA interacting with p27 in *Cks1*^*-/-*^ primary hippocampal neurons ( *n* = 9). ( *B7* ) Amount of active RhoA in *Cks1*^*-/-*^ primary hippocampal neurons ( *n* = 9).

**Figure 6b.**
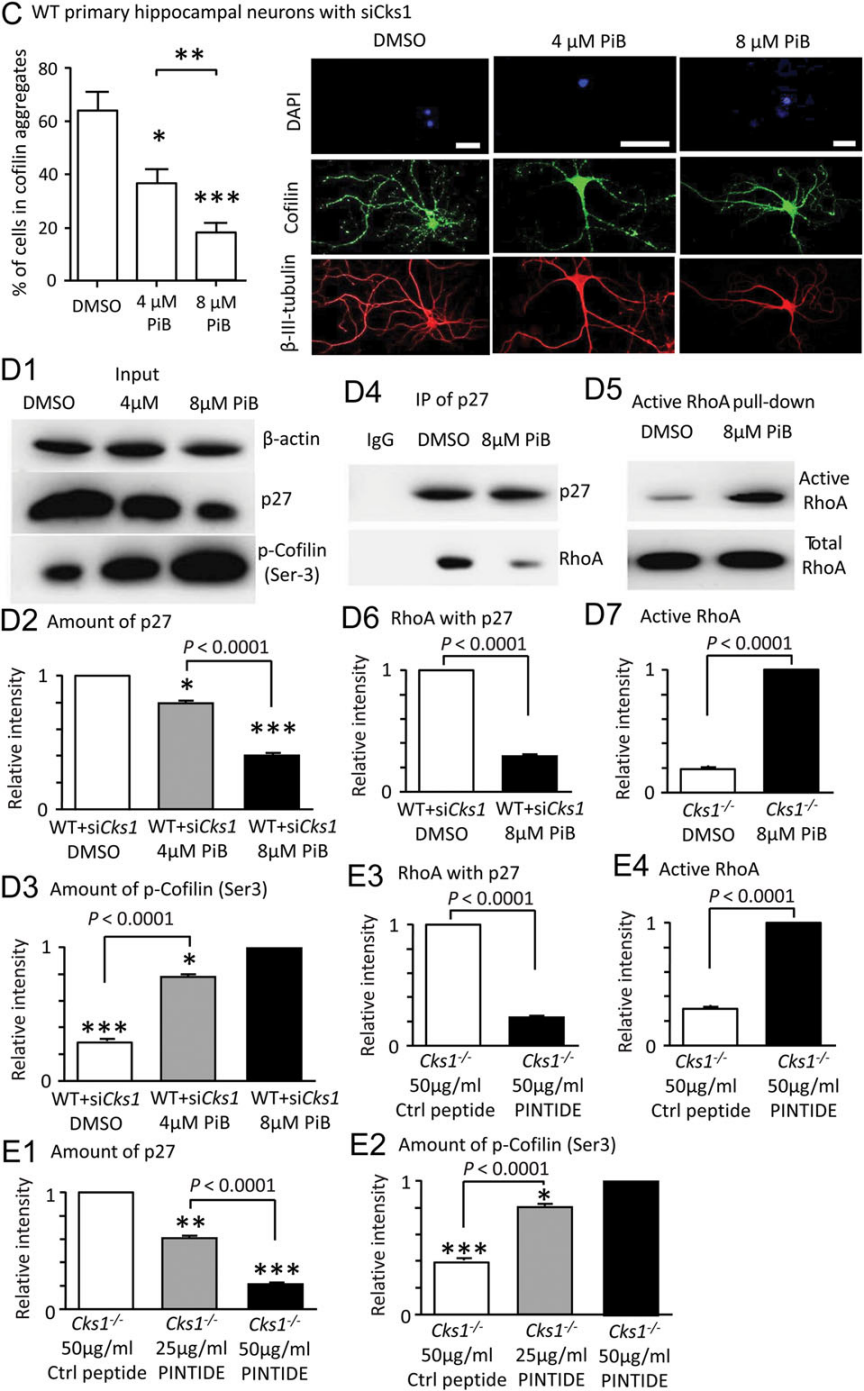
( *C* ) PiB treatment effectively reduced cofilin aggregates in WT primary hippocampal neurons treated with si *Cks1* (* *P* <0.05, ** *P* <0.01, *** *P* <0.001, 1-way ANOVA with Tukey's test). Confocal images showing cofilin and tubulin staining as in ( *A* ) *.* Scale 50 μm. ( *D* ) Similarly, in WT primary hippocampal neurons with *Cks1* knocked down by siRNA, ( *D1–D3* ) the PiB treatment decreased p27 level and increased phosphorylation of cofilin ( *D4–D7* ) via controlling the activity of RhoA, causing less RhoA binding with p27 and more active RhoA in PiB treated cells. Data were expressed as mean ± SEM ( *n* = 9).

**Figure 7.**
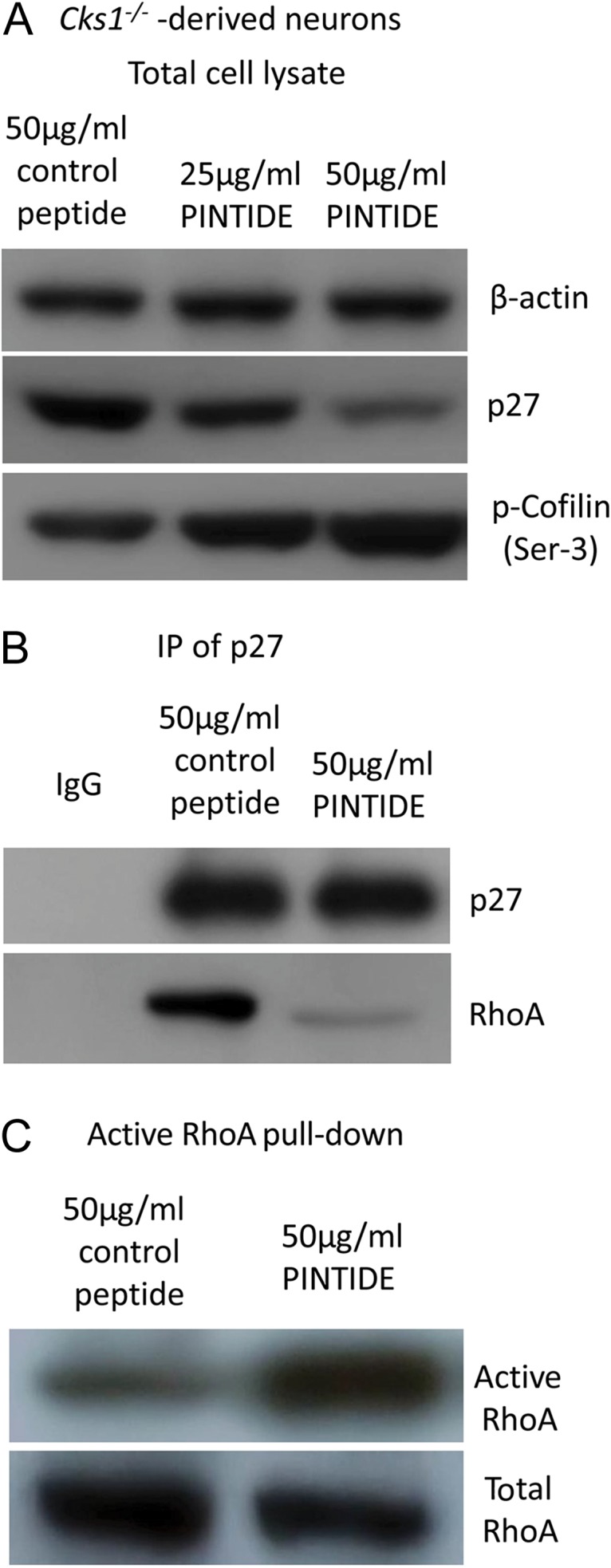
PIN1 inhibitory phosphopeptide decreased p27 levels and activated RhoA in Cks1 ^−/−^ neurons. In *Cks1*^−/−^ -derived neurons treatment of PIN1 inhibitory phosphopeptide (PINTIDE) ( *A,B* ) decreased the p27 levels, and increased cofilin phosphorylation and ( *C* ) increased activated RhoA, demonstrated with immunoprecipitation, GST-rhotekin pull-down, and immunoblotting.

## Discussion

CKS proteins are generally regarded as cell-cycle regulators. Although CKS1 does not bind the brain specific CDK, CDK5 directly ( Pines 1996 ), it may bind indirectly as a complex in the brain ( Veeranna et al. 2000 ). Here we describe a post-mitotic role for CKS1 in facilitating dendritic spine maturation in the hippocampus. We showed that CKS1 is actively expressed in the adult brain. *Cks1*^−/−^ mice exhibit impaired learning of hippocampus-dependent tasks, implying that CKS1 is required for the establishment of memory. Indeed, electrophysiological studies showed that the absence of CKS1 seriously compromised establishment of LTP.

In our model, we ascribe the phenotypes we saw in the *Cks1*^−/−^ mice to decreased RhoA kinase activity due to increased binding to p27. In order to establish long-lasting LTP, inactivation of cofilin by Rho kinase-mediated phosphorylation is required to increase F-actin content within dendritic spines ( Fukazawa et al. 2003 ). RhoA, through its effector RhoA kinase ROCK, activates LIM kinase (LIMK) that in turn phosphorylates cofilin ( Maekawa et al. 1999 ). Accordingly, a number of chemical agents, which interfere with actin polymerization, specifically block late LTP ( Rex et al. 2009 ). In the context of the hippocampus, we postulate that *Cks1*^−/−^ partially phenocopies the S3A cofilin mutant. The cofilin S3A mutant is non-phosphorylatable on serine 3, hence resistant to inhibitory phosphorylation. Over-expression of the S3A mutant results in an increase in the active form of cofilin and an inability to mature dendritic spines to the mushroom form. Also, elevated cofilin activity under certain conditions has been shown to contribute to enhanced AMPA receptor trafficking during synaptic potentiation ( Gu et al. 2010 ). This may explain the increased amplitude seen in the *Cks1*^−/−^ background. Of note, cofilin phosphorylation is decreased, but not absent in the *Cks1*^−/−^ mouse. This is expected, as cofilin phosphorylation is under control of multiple signaling pathways ( Pontrello and Ethell 2009 ). A study has showed that p27 promotes microtubule polymerization and negatively regulates myosin II activity ( Godin et al. 2012 ). Inhibition of myosin IIb has been shown to destabilize mushroom spines and inhibit excitatory synaptic transmission ( Ryu et al. 2006 ; Koskinen et al. 2014 ). Further investigation is required to see whether this also contributes to the *Cks1*^−/−^ phenotype.

The implication of CKS1 in memory formation is manifold. First, this suggests an evolution redundancy in the use of cellular mechanisms that control cytoskeleton remodeling within and without the mitotic cycle. Secondly, this implies that when neurons exit the mitotic cycle, certain components of the cell-cycle machinery remain, but take on different roles. Cyclin E has been shown to play a similar role in post-mitotic neurons ( Odajima et al. 2011 ). Our work therefore adds to the increasing repertoire of cell-cycle proteins that play non-cell-cycle dependent roles in neurons and in neurodegeneration ( van Leeuwen and Hoozemans 2015 ).

**Figure 8.**
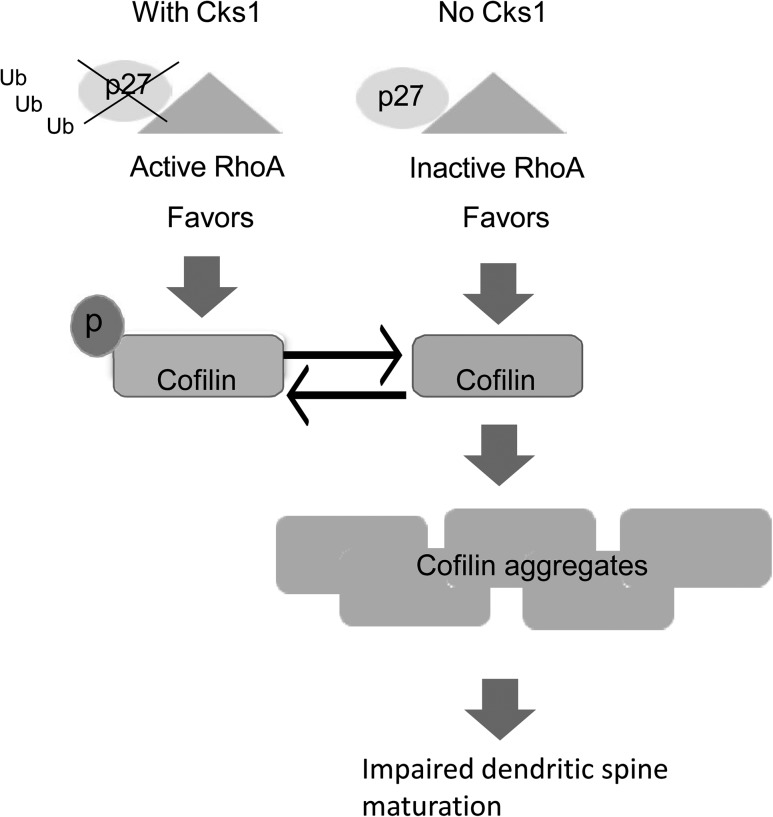
CKS1-mediated control of RhoA and cofilin phosphorylation via p27. Cks1, via regulating p27 ubiquitylation and degradation, fine tunes RhoA activity, and hence cofilin phosphorylation during this process. In the absence of Cks1, p27 level is increased. As a result, there is less active RhoA and decreased in cofilin phosphorylation, leading to formation of cofilin aggregation and impairment of dendritic spine maturation.

In summary, our results on this *Cks1*^−/−^ murine model demonstrate that CKS1 has a cell-cycle independent role in adult hippocampus contributing to memory consolidation, pyramidal cell dendritic spine maturation and late LTP. Inhibition of CKS1 facilitates formation of cofilin aggregates through the p27-RhoA axis (Fig. 8 ), yet, the exact role of CKS1 in the pathogenesis of human neurodegenerative diseases remains to be determined.

## Supplementary Material

Supplementary Data
