# Supplementary material for: Deficiency of Cks1 Leads to Learning and Long-Term Memory Defects and p27 Dependent Formation of Neuronal Cofilin Aggregates
Source: Cereb Cortex. 2016 Nov 19;27(1):11–23. doi: 10.1093/cercor/bhw354 (PMC5939225; doi:10.1093/cercor/bhw354)
Supplement: Supplementary Data [file supplementarytablewithlegend3.pdf]

Supplementary Table 1

	WT	Cks1-KO
**Body Position**		
0= Completely flat		
1= Lying on side		
2= Lying prone		
3= Sitting or standing	X	X
4= Rearing on hind legs		X
5= Repeated vertical leaping		
**Spontaneous Activity**		
0 = None, resting		
1 = Casual scratch, groom, slow movement	X	X
2 = Vigorous scratch, groom, moderate movement	X	X
3 = Vigorous, rapid/dart movement		X
4 = Extremely vigorous, rapid/dart movement		
**Respiration Rate**		
0 = Gasping, irregular		
1 = Slow, shallow		
2 = Normal	X	
3 = Hyperventilation	X	X
**Tremor**	0	0
**Transfer arousal**		
0 = Coma		
1 = Prolonged freeze, then slight movement		
2 = Extended freeze, then moderate movement		X
3 = Brief freeze, then active movement	X	X
4 = Momentary freeze, then swift movement		
5 = No freeze, immediate movement		
6 = Extremely excited ("manic")		
**Locomotor Activity**	6	8.7
**Palpebral Closure**	0	0
**Piloerection**	0	0
**Startle Response**	None	None
**Gait**	Normal	Normal
**Pelvic Elevation**	3mm	3mm
**Tail Elevation**		
0 = Dragging	X	
1 = Horizontally extended	X	X
2 = Elevated / Straub Tail	X	X
**Trunk Curl**	present	present
**Righting Reflex**	Normal	Normal
**Contact Righting Reflex**	present	present
**Provoked Biting**		
0 = Absent	X	
1 = Present	X	X

	WT	Cks1-KO
**Body Tone**		
0 = Flaccid, no return of cavity to normal		
1 = Slight resistance	X	X
2 = Extreme resistance, board like	X	X
**Pinna Reflex**		
0 = None	X	X
1 = Active retraction, noderately brisk flick	X	X
2 = Hyperactive, repetitive flick		
**Corneal Reflex**		
0 = None		
1 = Active single eye blink	X	X
2 = Multiple eye blink	X	X
**Toe Pinch**		
0 = None		
1 = Slight withdrawal	X	X
2 = Moderate withdrawal, not brisk	X	X
3 = Brisk, rapid withdrawal	X	X
4 = Very brisk repeated extension and flexion		
**Wire Manoeuvre**		
0 = Active grip with hindlegs	X	X
1 = Difficulty to grasp with hindlegs	X	X
2 = Unable to grasp with hindlegs		
3 = Unable to lift hindlegs, falls within seconds		
4 = Falls immediately		
**Skin colour**	Pink	Pink
**Heart Rate**		
0 = Slow, bradycardia		
1 = Normal	X	
2 = Fast, tachycardia	X	X
**Limb Tone**		
0 = No resistance		
1 = Slight resistance	X	X
2 = Moderate resistance		X
3 = Marked resistance		X
4 = Extreme resistance		
**Abdominal Tone**		
0 = Flaccid, no return of cavity to normal		
1 = Slight resistance	X	X
2 = Extreme resistance, board like		X
**Salivation**	None	None
**Defaecation**	P	P
**Weight**	32g	23.57g

	WT	Cks1-KO
**Fear**	None	None
**Aggression**		
0 = None	X	
1 = Provoked biting or attack	X	X
**Vocalization**		
0 = None	X	
1 = Provoked during handling	X	X
**Body Temperature**	38.68	39.27
**Irritability**		
0 = None		
1 = Struggle during supine restraint	X	X
**Limb Grasping**	present	present
**Visual Placing**		
0 = None		
1 = Upon nose contact		
2 = Upon vibrasse contact		
3 = Before vibrasse contact (18mm)	X	X
4 = Early vigorous extension (25mm)		
**Grip Strength**		
0 = None		
1 = Slight grip, semi-effective		
2 = Moderate grip, effective	X	X
3 = Active grip, effective	X	X
4 = Unusually effective		
**Negative Geotaxis**		
0 = Turns and climbs the grid	X	X
1 = Turns but then freezes		
2 = Moves, but fails to turn		
3 = Does not move within 30 seconds		
4 = Falls off		
**Touch Escape**		
0 = No response		
1 = Mild (escape response to firm stroke)	X	X
2 = Moderate (rapid response to light stroke)	X	X
3 = Vigorous (escape response to approach)		
**Positional Passivity**		
0 = Struggles when held by tail	X	X
1 = Struggles when held by neck		
2 = Struggles when laid supine (on back)		
3 = Struggles when held by hind legs		
4 = No struggle		
**Urination**	N (5)/p(2)	N(4)/P(3)

Supplementary Table 1 SHIRPA protocol
First-line phenotyping screening of wild-type and Cks1-/- littermates (3 males and 3 females each, aged 12 weeks) using the modified SHIRPA protocol (details of the protocol is found at http://empress.har.mrc.ac.uk/browser/?sop_id=10_002_0.)
“X” denotes animals scoring under each category.
